# Genomic analysis of extended-spectrum beta-lactamase (ESBL) producing *Escherichia coli* colonising adults in Blantyre, Malawi reveals previously undescribed diversity

**DOI:** 10.1099/mgen.0.001035

**Published:** 2023-06-14

**Authors:** Joseph M. Lewis, Madalitso Mphasa, Rachel Banda, Mathew A. Beale, Jane Mallewa, Catherine Anscome, Allan Zuza, Adam P. Roberts, Eva Heinz, Nicholas R. Thomson, Nicholas A. Feasey

**Affiliations:** ^1^​ Malawi-Liverpool Wellcome Research Programme, Kamuzu University of Health Sciences, Blantyre, Malawi; ^2^​ Department of Clinical Sciences, Liverpool School of Tropical Medicine, Liverpool, UK; ^3^​ Department of Clinical Infection, Microbiology and Immunology, University of Liverpool, Liverpool, UK; ^4^​ Wellcome Sanger Institute, Hinxton, UK; ^5^​ Kamuzu University of Health Sciences, Blantyre, Malawi; ^6^​ Department of Tropical Disease Biology, Liverpool School of Tropical Medicine, Liverpool, UK; ^7^​ London School of Hygiene and Tropical Medicine, London, UK

**Keywords:** Africa south of the Sahara, Drug resistance, microbial, Extended-spectrum beta-lactamase, Whole-genome sequencing

## Abstract

*
Escherichia coli* is one of the most prevalent Gram-negative species associated with drug resistant infections. Strains that produce extended-spectrum beta-lactamases (ESBLs) or carbapenemases are both particularly problematic and disproportionately impact resource limited healthcare settings where last-line antimicrobials may not be available. A large number of *E. coli* genomes are now available and have allowed insights into pathogenesis and epidemiology of ESBL *E. coli* but genomes from sub-Saharan Africa (sSA) are significantly underrepresented. To reduce this gap, we investigated ESBL-producing *E. coli* colonising adults in Blantyre, Malawi to assess bacterial diversity and AMR determinants and to place these isolates in the context of the wider population structure. We performed short-read whole-genome sequencing of 473 colonising ESBL *E. coli* isolated from human stool and contextualised the genomes with a previously curated multi-country collection of 10 146 *E. coli* genomes and sequence type (ST)-specific collections for our three most commonly identified STs. These were the globally successful ST131, ST410 and ST167, and the dominant ESBL genes were *bla*_CTX-M_, mirroring global trends. However, 37 % of Malawian isolates did not cluster with any isolates in the curated multicountry collection and phylogenies were consistent with locally spreading monophyletic clades, including within the globally distributed, carbapenemase-associated B4/H24RxC ST410 lineage. A single ST2083 isolate in this collection harboured a carbapenemase gene. Long read sequencing demonstrated the presence of a globally distributed ST410-associated carbapenemase carrying plasmid in this isolate, which was absent from the ST410 strains in our collection. We conclude there is a risk that carbapenem resistance in *E. coli* could proliferate rapidly in Malawi under increasing selection pressure, and that both ongoing antimicrobial stewardship and genomic surveillance are critical as local carbapenem use increases.

## Data Summary

All data and code to replicate this analysis are available as the *blantyreESBL* v1.4 R package (https://doi.org/10.5281/zenodo.5554081) available at https://github.com/joelewis101/blantyreESBL. Reads from all isolates sequenced as part of this study have been deposited in the European Nucleotide Archive, under PRJEB26677, PRJEB28522 and PRJEB36486 (short reads) and PRJNA869071 (Nanopore reads and hybrid assemblies). Accession numbers (as well as accession numbers of publicly available genomes used in this analysis) linked to sample metadata are provided in the R package, as supplementary data to this manuscript and on Figshare https://doi.org/10.6084/m9.figshare.22561738.v1 [1].

**Impact Statement**

Antimicrobial-resistant *Escherichia coli* producing extended-spectrum beta lactamase (ESBL) or carbapenemase enzymes have been identified by the World Health Organisation as priority pathogens of global concern, and disproportionately affect low-resource settings. In Blantyre, Malawi, 31 % of *E. coli* are now ESBL producers; the antimicrobial treatment of choice – carbapenems – are at best sporadically available and hence many of these isolates are locally untreatable. Whilst whole genome sequencing has provided some insight into mechanisms of virulence, antimicrobial resistance and spread of high-risk ESBL/carbapenemase *E. coli* clones globally, studies analysing large numbers of *E. coli* often opportunistically examine isolate collections from diagnostic services in high-income settings. Understanding how genomic epidemiology of ESBL *E. coli* in sub-Saharan Africa differs is essential to provide insight into global drivers of transmission and for regional policy makers. We therefore sequenced 473 ESBL-producing *E. coli* genomes colonising adults in Blantyre, Malawi. We analyse determinants of antimicrobial resistance and virulence and place the isolates in wider context using a previously published global *E. coli* collection that was generated to represent the whole species diversity of sequences publicly available. We find that there is diversity in our ESBL-producing isolates from Malawi not reflected in the curated global collection: widely successful antimicrobial-resistance associated *E. coli* sequence types are represented in Blantyre, but locally circulating subclades are apparent. A lack of comparator genomes from surrounding countries and sub-Saharan Africa in general means that the full geographic distribution of these subclades remains unknown. We find that carbapenemases in our collection are unusual but present and carried on globally disseminated plasmids. So too are globally successful *E. coli* sequence types that in other settings have stably carbapenemase-associated lineages. Although the isolates we sequenced from Malawi typically lacked carbapenemases, carbapenem use is increasing in Malawi and their unstewarded use will accelerate selection for carbapenemases in *E. coli* in the future. Our study highlights the need for robust antimicrobial stewardship protocols as these agents are introduced, and the need for local, regional and continental genomic surveillance to track the spread of AMR and inform prevention efforts.

## Introduction

*Escherichia coli* is a ubiquitous bacterium, a human gut commensal and common pathogen [2]. Beta-lactam antibiotics (including third generation cephalosporins, 3GC) are widely used for treatment of Gram-negative infections like *E. coli* but are largely rendered ineffective by bacteria expressing extended-spectrum beta lactamase (ESBL) enzymes. ESBL-producing bacteria have disseminated globally, in many cases leaving carbapenems as the only clinically effective and well tolerated treatment option [3, 4]. These agents are now also under threat given the increasing spread of strains producing carbapenem-inactivating carbapenemase enzymes, and *E. coli* producing ESBL and carbapenemase enzymes have been identified as priority pathogens by the World Health Organisation [5]. Global genomic surveillance has provided insight into the mechanisms and epidemiology of their spread, suggesting that capture of virulence and AMR determinants via horizontal gene transfer by so-called high risk clones results in fitness and/or colonisation advantages and subsequent global dissemination [6]. This phenomenon is well described in *E. coli* sequence type (ST) 131 [7], associated with the ESBL-encoding gene *bla*_CTX-M-15_, but has also been recently described in other carbapenemase-associated *E. coli* lineages, such as ST167 [8] and ST410 [9].

Whole genome sequencing efforts to date have largely focussed on AMR *E. coli* collections from high-income settings [10], however the greatest burden from drug-resistant infection is in low- and middle-income countries (LMIC), particularly in sub-Saharan Africa [11, 12]. The genomic epidemiology of AMR *E. coli* in these regions is poorly described. The 3GC antimicrobial ceftriaxone has been widely used in Malawian hospitals since its introduction to the national formulary in 2005 [13], but carbapenem use is not yet routine. Since 2005, ESBL-producing *E. coli* have become an increasing problem in clinical practice and now represent 31 % of invasive *E. coli* in Blantyre [14], whereas carbapenem resistance has so far only been described sporadically [15]. There is a significant need for better access to carbapenem antimicrobials to treat resistant infections, but the example of ceftriaxone in Malawi and of carbapenems globally shows that carbapenem resistance will likely disseminate rapidly as carbapenem use increases. In this context, both robust antimicrobial stewardship and ongoing genomic surveillance are critical.

In this study, we analysed the genomic diversity of ESBL-producing *E. coli* collected from a study of gut mucosal colonisation with ESBL Enterobacterales in Blantyre, Malawi, as carriage typically precedes invasive disease. We describe the diversity and AMR determinants of ESBL *E. coli *. We contextualised genomic data from Blantyre with that from large public datasets to understand the diversity of colonising *E. coli* in our setting and to gain insights into how well high-income country (HIC)-focused collections capture and represent the genetic diversity of our ESBL-producing carriage isolates from a low-income country.

## Methods

The clinical study which provided the isolates for this analysis was approved by the Liverpool School of Tropical Medicine Research Ethics Committee (16-062) and University of Malawi College of Medicine Research Ethics Committee (COMREC P.11/16/2063). The isolates analysed in this study were selectively cultured from stool and rectal swabs collected from adults in Blantyre, Malawi, as part of a study of longitudinal carriage of ESBL-producing Enterobacterales, as previously described [16]. Briefly, three groups of adults (≥ 16 years) were recruited following informed consent to participate in the study: i) 225 adults with sepsis, empirically treated with antibiotics in the emergency department of Queen Elizabeth Central Hospital (QECH), Blantyre, Malawi; ii) 100 antimicrobial-unexposed adult inpatients at QECH; and iii) 100 antimicrobial-unexposed adults recruited from the community. Antimicrobial unexposed was defined as no receipt of antimicrobials in the previous 4 weeks, with the exception of long-term co-trimoxazole preventative therapy (CPT, trimethoprim-sulfamethoxazole administered lifelong to people living with HIV in Malawi as per World Health Organisation [WHO] guidelines) [17] or antituberculous chemotherapy (usually comprising rifampicin, isoniazid, pyrazinamide and ethambutol). Up to five stool samples (or rectal swab samples) were collected over the course of 6 months and aerobically cultured overnight at 37 °C on ChromAGAR ESBL-selective chromogenic media (ChromAGAR, France) before being speciated with the API system (BioMériuex, France).

A subset of isolates identified as *E. coli* underwent DNA extraction and sequencing: one *E. coli* colony pick from the first 507 samples where *E. coli* was identified (this number determined by logistic considerations). DNA was extracted from overnight nutrient broth cultures using the Qiagen DNA mini kit (Qiagen, Germany) as per the manufacturer’s instructions. DNA was sequenced at the Wellcome Sanger Institute on the Illumina HiSeq X10 instrument (Illumina Inc., United States) to produce 150 bp paired end reads. Species was confirmed with Kraken v0.10.6 and Bracken v1.0 [18] with a 8 Gb MiniKraken database (3 April 2018). We first reconstructed a core gene phylogeny for the study isolates: *de novo* assembly was performed using SPAdes v3.14.0 [19] and the pipeline described by Page *et al*. [20] and quality of the assemblies assessed with CheckM v1.1.2 [21] and QUAST v5.0.2 [22]. Assemblies with a total assembled length of <4 Mb or with a CheckM-defined contamination of ≥10 % were excluded from further analysis. Included assemblies had a median 92 (IQR 68–122) contigs and N50 of 180kbp (IQR 123-234kbp). Assemblies were annotated with Prokka v1.5 using a genus-specific database from RefSeq [23] and the Roary v1.007 pangenome pipeline [24] used to identify core genes with a blast threshold of 95 %; paralogs were not split. Genes present in ≥99 % samples were considered core, and a pan-genome of 26 840 genes was determined, of which 2966 were core. The core genes were concatenated to a 1 388 742 base pseudosequence; 99 693 variable sites were identified and extracted with snp-sites v2.4.1 [25] -c to select for ACGT-only sites and a maximum-likelihood phylogeny inferred from this alignment with IQ-TREE v1.6.3 [26]. The ModelFinder module was used to select the best fitting nucleotide substitution model: the general time reversible model with FreeRate site heterogeneity with five parameters, which was fitted with 1000 ultrafast bootstrap replicates.

ARIBA v.2.12.1 [27] was used on the reads to identify AMR-associated genes from the SRST2 curated version of the ARG-ANNOT database [28], and was also used to call single nucleotide polymorphisms (SNPs) in the quinolone-resistance determining regions (QRDR) *gyrA, gyrB, parC* and *parE*, using the wild-type genes from *Escherichia coli* K-12 substr. MG1655 (NC_000913.3) as reference. QRDR mutations were filtered to include only those which are associated with quinolone resistance in *E. coli* in the comprehensive antibiotic resistance database [29] (CARD). Beta lactamases were phenotypically classified according to https://ftp.ncbi.nlm.nih.gov/pathogen/betalactamases/Allele.tab. Throughout this manuscript we present prevalence of AMR-associated genes rather than inferred resistance. ARIBA was also used to determine *E. coli* multilocus sequence type (ST) as defined by the seven-gene Achtman scheme [30] hosted at pubMLST (https://pubmlst.org/), to identify plasmid replicons using the PlasmidFinder database [31], and to determine pathotype by identifying genes contained in the VirulenceFinder database [32]. Pathotype was assigned based on the criteria in Table S1, available in the online version of this article [33]. *E. coli* phylogroups were defined by the Clermont scheme using ClermonTyping v20.3 [34].

To place the isolates from this study in context of the wider *E. coli* population structure, we used a dataset from a previously described highly curated multi-country collection of *E. coli* genomes comprising 10 146 *E. coli* genomes from around the world [10]. This collection was extremely thoroughly quality-controlled, resulting in a curated set of genomes representing a species-wide background dataset that we used to place our samples in a global context. Henceforth this will be referred to as the global collection. It predominantly included samples collected in Europe (*n*=7608, 75 %) and the USA (*n*=1654, 16 %), whereas samples from Africa (*n*=264, 3 %), Asia (*n*=165, 2 %), South America (*n*=89, 1 %) and Oceania (*n*=30, < 1 %) were unusual. Clinical source of isolation was available for 39 % of genomes (*n*=3921; of these, 45 % (*n*=1718) were recorded as isolated from blood, 6 % (*n*=574) from urine, <1 % (*n*=20) as wound/respiratory and 41 % (*n*=1600) as faeces or rectal swab. In the original publication describing this collection, the 10 146 genomes were clustered with the popPUNK algorithm into 1154 lineages and ten genomes from each of the largest 50 lineages were selected to produce a collection of 500 genomes representative of these largest lineages. To place our isolates in context with this collection we first used popPUNK v1.1.5 [35] to compare our assemblies with the popPUNK database of all 10 146 genomes from the global collection. This allowed us to assign genomes from Malawi into the respective published popPUNK groups and compare the distribution to the global collection. To assess the effect of our sampling strategy (ESBL producing *E *. coli from stool) on phylogroup distribution, we stratified the global collection by presence of ESBL/carbapenemase/ampC gene and source of isolation (‘invasive’ versus ‘stool’): we used ARIBA as described above to infer presence of ESBL/carbapenemase/ampC and grouped isolates from blood, urine, wound swab and respiratory sources as ‘invasive’, and samples from faeces or rectal swab as ‘stool.’

We then constructed a core-gene phylogeny. We combined the 500 curated, representative assemblies of the 50 largest lineages from the global collection with up to ten genomes from all other PopPUNK-defined lineages in the collection; if a lineage included ≤10 genomes we included all of them, otherwise we randomly sampled ten genomes from the lineage to include. In total, this left a dataset of 2776 genomes. We added a further 97 genomes from a previous study of *E. coli* isolated from blood at QECH, where archived samples were selected for sequencing to maximise temporal and antimicrobial susceptibility profile diversity [36]. See Figure S1 for flowchart of included isolates. QC, assembly, determination of ST and phylogroup of these samples proceeded as described above. Following QC, four genomes from this latter study were excluded, and combined with our 473 new Blantyre genomes, this left a final working dataset of 3342 *E. coli* genomes for analysis. We used the Roary pan-genome pipeline to infer a pangenome, identifying 73 062 gene orthologs in this collection, of which 2182 were core and formed a concatenated sequence of 530 659 bases with 53 410 variable sites. These were extracted with snp-sites and used to infer the phylogeny, using IQ-TREE with the same substitution model as above, and again with 1000 ultrafast bootstrap replicates.

To better describe the phylogeny of the three most common STs in our dataset, ST131, ST410 and ST167 in greater resolution, we inferred lineage-specific phylogenies using a map-to-reference approach, contextualised with publicly available genomes. We used previously curated multi-country collections of 862 ST131 genomes [37], 327 ST410 genomes [9], and 181 ST167 genomes [38]. For all isolates, we obtained available raw reads (*n*=108 genomes for ST167, *n*=327 for ST410 and *n*=862 for ST131), performed QC with fastQC v0.11.8 (https://www.bioinformatics.babraham.ac.uk/projects/fastqc/) and multiqc v1.8 [39], trimmed raw reads with Trimmomatic v0.39 [40], removing adapter sequences and leading or trailing bases with phred score <4, bases with a mean score <20 (over a sliding window of four bases), and any reads with length below 36 following removal of low quality bases. We mapped the reads to ST specific references: for ST131 the ST131 NCTC 13441 reference strain from the UK Health Security Agency culture collection (NCBI accession NZ_LT632320.1) and for ST167 and ST410 reference genomes from the curated FDA-ARGOS database [41] (GenBank accession CP023870.1 for ST167 and CP023870.1 for ST410). We used the snippy v4.6.0 [42] pipeline with default settings, excluding mapped pseudosequences with mean mapped coverage depth <20× from further analysis. Following this additional control; 102 ST167, 326 ST410, and 855 ST131 context genomes were retained for further analyses, and combined with the 38 ST167, 45 ST410 and 64 ST131 from this study. Areas of recombination were predicted with Gubbins v3.0.0 [43] and masked. The variable sites remaining (13 693 sites in a 4 711 093-base alignment for ST410, 6119 sites in 4 897 877 bases for ST167 and 18 171 sites in 5 174 631 bases for ST131) were used to reconstruct a phylogeny with IQ-TREE as above. Presence of AMR genes and plasmid replicons were inferred as above.

To gain better insights into carbapenemase associated strains in our setting we performed long read sequencing on a *bla*_NDM-5_ carbapenemase associated ST2083 isolate (the only carbapenemase-containing isolate in our collection) and a randomly selected ST410 isolate. This ST was chosen because ST410 are a global disseminated stably *bla*_NDM-5_ associate lineage, but Malawian ST410 isolates (in this study) all lacked the expected carbapenemase genes (see Results below). We isolated long-fragment DNA using the epicentre MasterPure Complete DNA and RNA Purification Kit as per manufacturer instructions, performed long-read sequencing using a MinION sequencer (Oxford Nanopore) and generated hybrid assemblies. We assembled our genomes using unicycler [44] v0.4.7, using recommended settings for hybrid assembly of Illumina and nanopore reads. Annotation was performed using Prokka as above. To map reads back to the assembly for investigating coverage of AMR regions and ST-specific SNPs in ST410, we used snippy with the default settings. Analysis of the conserved plasmids was performed using BRIG v0.95 [45] with the plasmids CP034954.1, CP034955.1, CP034956.1 and CP034957.1 from the *bla_NDM-5_*-encoding ST410 strain SCEC020026 [9] as reference sequences. The unicycler-assembled ST410 strain resulted in nine contigs (4 670 094 bp; 127 483 bp; 98 229 bp; 33 870 bp; 2 088 bp; 245 bp; 194 bp; 165 bp; 119 bp); we did not include the chromosome (largest fragment) and the smallest four plasmids in the comparison as they are likely spurious assemblies; sequences below 200 bp furthermore get removed by default by genbank whole-genome upload as likely contaminants. The unicycler-assembled ST2083 strain resulted in five contigs with lengths (4 967 815 bp; 110 151 bp; 108 044 bp; 48 551 bp; 46 161 bp), all of which were included in the BRIG plasmid comparison except for the chromosome (largest fragment). The assembled ST410 and ST2083 (including all small fragments) and their respective long reads used for the hybrid assembly are deposited under sample accessions SAMN30282368 and SAMN30282369, respectively.

All statistical analyses were carried out in R v4.1.1 (R Foundation for Statistical Computing, Vienna, Austria) and trees were visualized using the *ggtree* v2.2.4 [46] package. Summary statistics, where presented, are medians and interquartile ranges or proportions unless otherwise stated. Short reads from all isolates sequenced in this study have been deposited in the European Nucleotide Archive under project IDs PRJEB26677, PRJEB28522 and PRJEB36486. All data and code to replicate this analysis are available as the *blantyreESBL* v1.4 [47] R package available at https://joelewis101.github.io/blantyreESBL/. Sample accession numbers linked to sample metadata as well as accession numbers of all context genomes used in this analysis are available as part of the R package and as supplementary data to this manuscript.

## Results

### Population structure of ESBL-producing *E. Coli* from Malawi

**Fig. 1.**
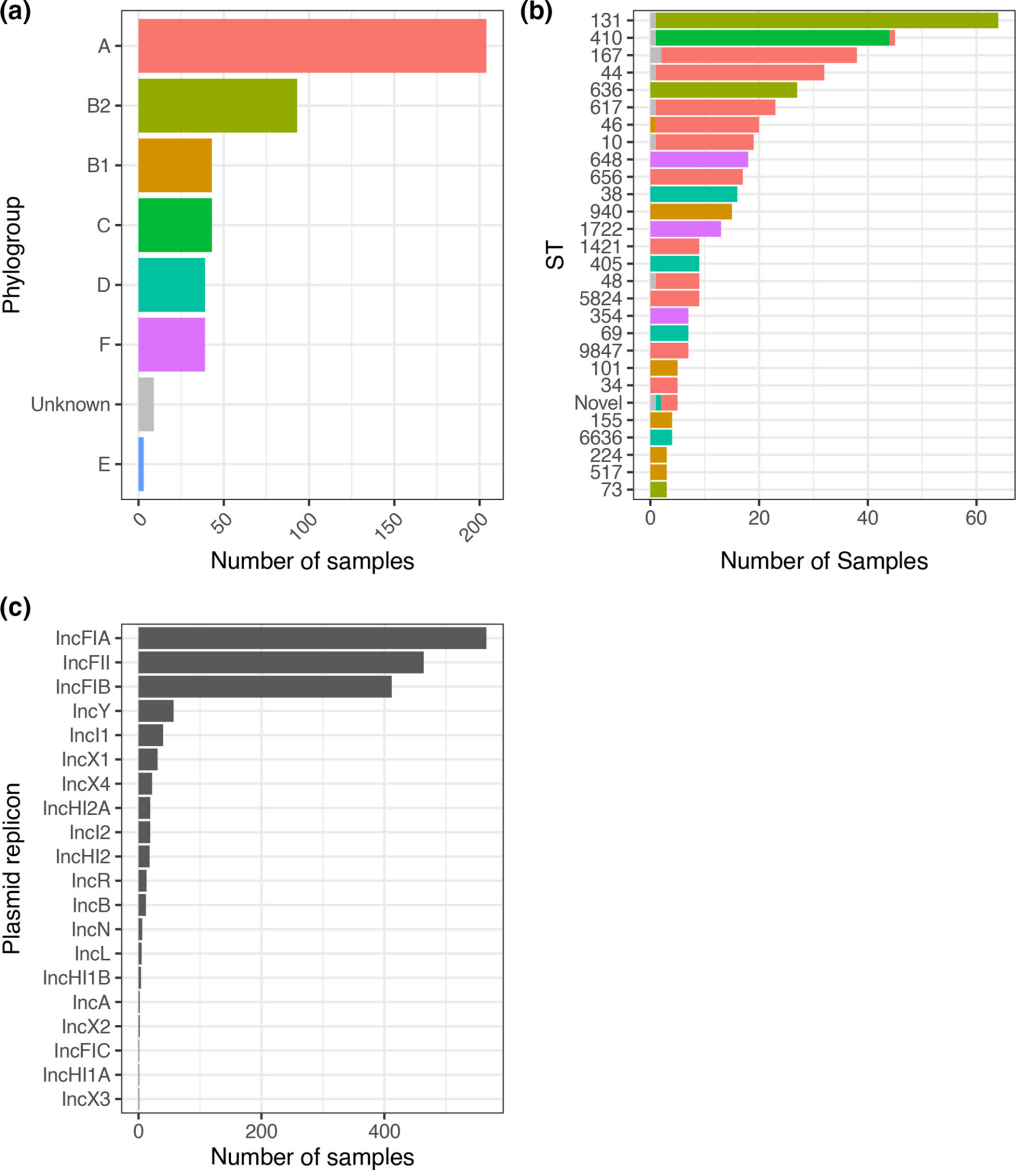
Phylogroup (**a**) and sequence type (**b**) distribution of included isolates and (**c**) plasmid Incompatibility-type plasmid replicons identified.

Following quality control, 473 ESBL *E. coli* genomes sequenced for this study were included in the analysis, 440 from participants enrolled in hospital, and 33 from community members, with a median 2 (IQR 1–5) samples per participant. This represents 64 % (473/641) of all *E. coli* identified by culture in the parent study; a full description of study participants and temporal trends has previously been made [16]; ESBL *E. coli* carriage prevalence was more common in hospitalised participants, which explains the preponderance of genomes from this cohort in this analysis. Briefly, the 473 isolates were recovered from 230 participants but within participant diversity was considerable: in 57 % of participants with more than one sample (*n*=131/230 patients, median 2 samples/patient [range 2–5]), the same ST was isolated only once (Figure S2A), and the distribution of pairwise core-genome SNP differences was similar between samples of the same ST whether sample pairs were within- or between-participants. On this basis, we included all samples in further analysis (Figure S2B). The most common phylogroup was A (43 %), followed by phylogroup B2 (20 %), B1 (9 %), C (9 %), F (8 %) and D (5 %), with nine samples untyped by the Clermont scheme (Fig. 1a). Fifty-seven recognised STs were identified in our isolates, with a median of 2 (IQR 1–9, range 1–64) samples per ST (Fig. 1b). Examining the core gene tree topology, we confirmed that these STs were largely monophyletic (Figure S3). The three most frequent STs accounted for 32% of isolates: ST131 (64/473 [14 %] of isolates) followed by ST410 (45/473 [10 %]) and ST167 (38/473 [8 %]).

We next placed the ESBL-producing carriage isolates from this study in context of the wider species diversity using the global collection [10]. As the global collection is an amalgamation of genomes from studies with different sampling frames, we first examined phylogroup distributions of our ESBL-producing carriage collection stratified by presence of genes encoding ESBL/ampC/carbapenemase enzymes and source of isolation (stool vs invasive, Figure S4). Out of 10 146 isolates in total, 3921 contained the necessary metadata information, and within this subselection of 576 isolates encoding ESBL/ampC/carbapenemases (both invasive and from stool), 465/576 (82 %) were phylogroup B2, whilst phylogroup A was unusual (20/576, 3 %), in contrast to our isolates from Malawi. This was also the case for the 253 global ESBL/ampC/carbapenemase isolates from stool (231/253 [91 %] phylogroup B2, 5/252 [2 %] phylogroup A).

**Fig. 2.**
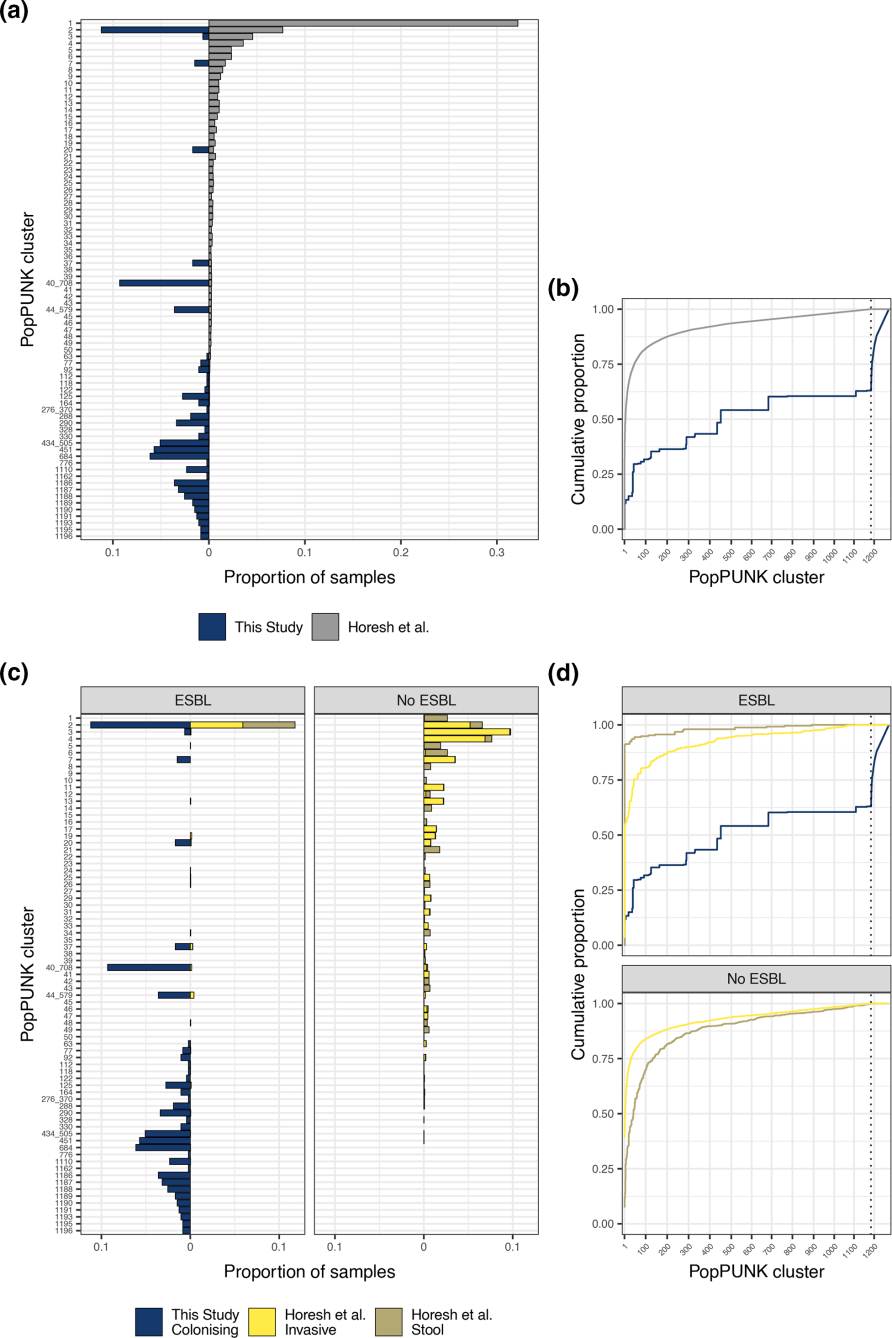
Comparing distribution of popPUNK clusters in Malawian and global collections. (a): proportion of samples assigned to a given popPUNK cluster in Malawian (left) and global (right) isolates. Clusters are arranged in name-numeric order which, by definition, is size order from the original publication from largest to smallest. Clusters 1–50 (accounting for 76 % of global isolates) are shown along with any cluster containing at least three Malawian isolates. (b): Cumulative proportion of samples with given cluster membership, stratified by study; clusters are again numerically ordered on x-axis. Dotted line shows the maximum cluster identifier that was defined in the global collection (*n*=1184); clusters with an identifier greater than 1184 (to right of dotted line) were not present in global collection. Clusters with an identifier made up of two numbers separated by an underscore are clusters that were two separate clusters in the original global collection but have been merged after Malawian genomes were added (e.g. 40_708). (c) and (d): same analysis, stratified by presence of ESBL/ampC/carbapenemase gene and source of isolation; for brevity, ESBL/ampC/carbapenemase is referred to as ‘ESBL’ in this plot.

Next, using popPUNK we assigned our 473 ESBL-producing carriage isolates to the clusters defined in the original description of the global collection: the algorithm assigned our isolates to 109/1154 of the original clusters (median size 1; IQR 1–3). The distribution of clusters differed between the isolates from this study and the subsampled global collection (Fig. 2, Table 1), and a large part of the diversity in PopPUNK cluster in our study was not represented in the global collection: 175/473 (37 %) of our isolates formed novel PopPUNK clusters unrelated to isolates in the global collection. Diversity in PopPUNK cluster between isolates in the global collection carrying genes encoding ESBL/ampC/carbapenemases was less than between those without (Fig. 2c, d). In particular, ESBL/ampC/carbapenemase isolated from stool in the global collection were overwhelmingly (230/253 [91 %]) members of PopPUNK cluster two (corresponding to ST131).

**Table 1.** Phylogroup, sequence type (ST), continent of collection and pathotype of popPUNK-defined clusters

Cluster	Phylogroup	This study		Horesh Collection			
		*n*	STs	*n*	STs	Location	Pathotype
2	B2	53	ST131 (1.00)	781	ST131 (0.99)	Europe (0.73); Unknown (0.12); Americas (0.11); Oceania (0.03); Asia (0.00)	ExPEC (0.54); Not determined (0.46)
40_708	C	44	ST410 (1.00)	29	ST23 (0.41); ST410 (0.34); ST2230 (0.07); ST369 (0.07); ST5491 (0.07); ST5286 (0.03)	Europe (0.62); Americas (0.31); Asia (0.07)	ExPEC (0.55); STEC (0.21); ETEC (0.17); Not determined (0.07)
684	A	29	ST44 (1.00)	1	ST44 (1.00)	Europe (1.00)	ExPEC (1.00)
451	B2	27	ST636 (1.00)	1	ST636 (1.00)	Americas (1.00)	ExPEC (1.00)
434_505	A	24	ST167 (0.92); ST10 (0.08)	3	ST10 (1.00)	Americas (0.67); Europe (0.33)	ExPEC (0.67); Not determined (0.33)
44_579	F	17	ST648 (1.00)	26	ST648 (1.00)	Europe (0.42); Unknown (0.38); Americas (0.19)	ExPEC (0.73); Not determined (0.27)
1186	A	17	ST656 (1.00)	0	–	–	–
290	A	16	ST46 (1.00)	2	ST46 (1.00)	Americas (1.00)	ExPEC (0.50); Not determined (0.50)
1187	B1	15	ST940 (1.00)	0	–	–	–
125	A	13	ST617 (0.92); ST4981 (0.08)	7	ST617 (1.00)	Americas (0.71); Europe (0.29)	ExPEC (0.57); Not determined (0.43)
1188	A	12	ST167 (1.00)	0	–	–	–
1110	F	11	ST1722 (1.00)	1	ST1722 (1.00)	Europe (1.00)	ExPEC (1.00)
288	A	9	ST5824 (1.00)	3	ST227 (1.00)	Americas (1.00)	Not determined (1.00)
20	B2	8	ST131 (1.00)	48	ST131 (0.96); ST5432 (0.02); ST5494 (0.02)	Europe (0.75); Americas (0.12); Unknown (0.10); Oceania (0.02)	ExPEC (0.71); Not determined (0.29)
37	D	8	ST405 (1.00)	27	ST405 (0.96); ST964 (0.04)	Europe (0.63); Americas (0.33); Asia (0.04)	ExPEC (0.81); Not determined (0.11); ETEC (0.04); STEC (0.04)
1189	A	8	ST1421 (1.00)	0	–	–	–
7	D	7	ST69 (1.00)	174	ST69 (0.94); ST106 (0.03)	Europe (0.83); Americas (0.12); Unknown (0.05)	ExPEC (0.79); Not determined (0.19); EAEC (0.02)
1190	A	7	ST9847 (1.00)	0	–	–	–
1191	D	6	ST38 (0.83); Novel (0.17)	0	–	–	–
92	A	5	ST10 (1.00)	10	ST10 (1.00)	Europe (0.60); Americas (0.40)	ExPEC (0.60); Not determined (0.40)
164	A	5	ST34 (1.00)	6	ST34 (1.00)	Africa (0.67); Europe (0.33)	EPEC (0.67); Not determined (0.33)
330	B1	5	ST101 (1.00)	2	ST101 (1.00)	Europe (0.50); Unknown (0.50)	Not determined (1.00)
1193	A	5	ST10 (1.00)	0	–	–	–

PopPUNK cluster two was also the most frequently identified in our isolates (*n*=53 isolates from this study, all ST131). PopPUNK cluster 40_708 (*n*=44 isolates from this study, all ST410) was also common in both our Malawi collection and was amongst the 50 largest clusters in the global collection, with 29 isolates; whilst all of the Malawi isolates in this cluster contained ESBL/ampC/carbapenemase-genes, none of the six global collection isolates from stool did. Other large clusters in our Malawi collection had very few representatives in the global collection: the third and fourth largest clusters from Malawi were lineage 684 (*n*=29 in this study, all phylogroup A ST44) and 451 (*n*=27 all phylogroup A ST636) with only one isolate each in the global collection (both global isolates were invasive but with and without ESBL/ampC/carbapenemase genes, respectively, Table 1).

**Fig. 3.**
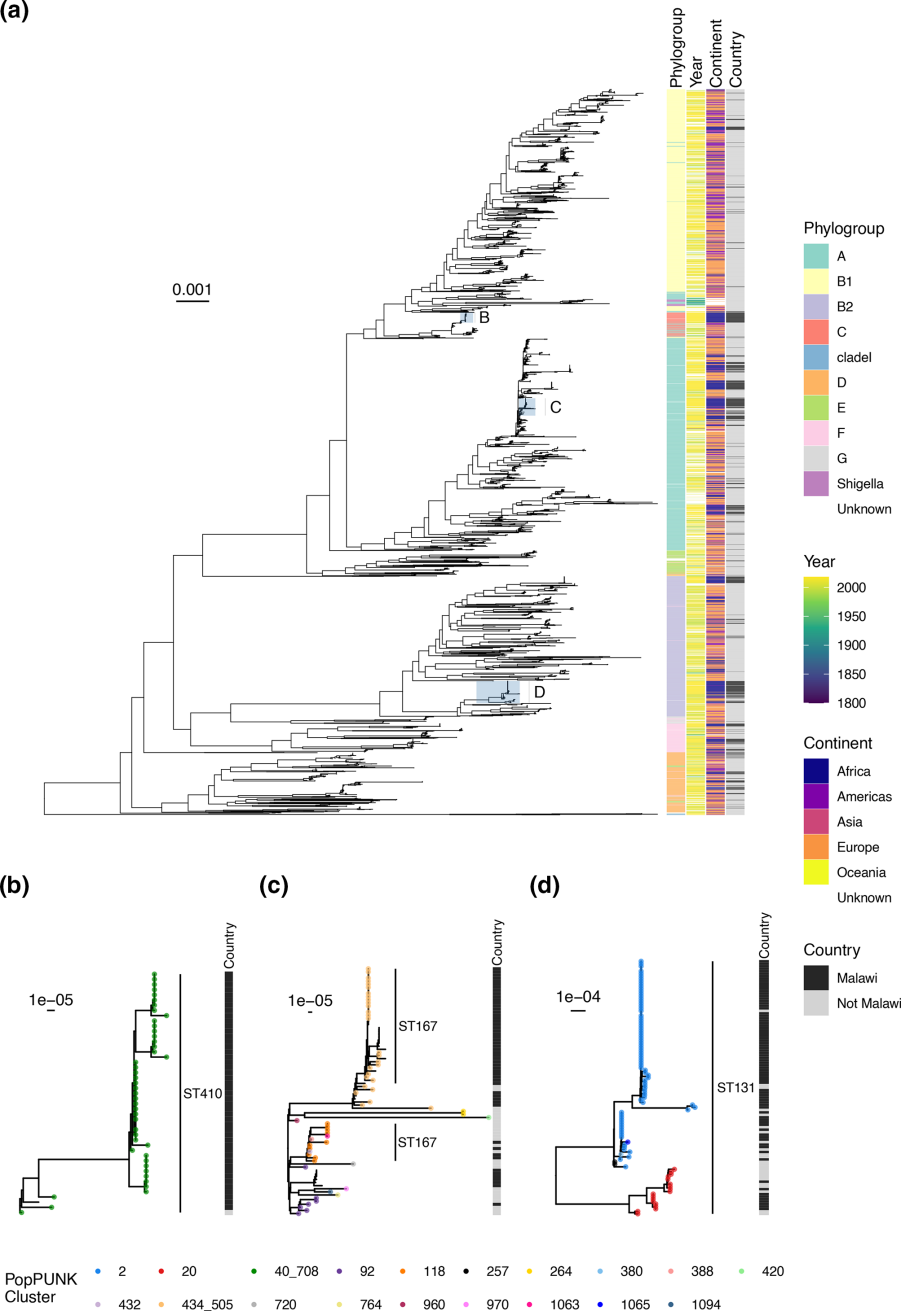
Midpoint rooted core-gene maximum likelihood phylogenetic tree of Malawian isolates with global context isolates (10 isolates each from the top 50 popPUNK clusters in the global collection, along with a further 92 Malawian isolates from a previous study), showing phylogroup, year of collection, continent, and country (Malawi vs not Malawi). Generally, Malawian isolates are distributed throughout the tree. (b–d) show magnified subtrees of the three most frequently identified STs: ST 410 (**b**), ST167 with surrounding phylogroup A isolates (**c**) and ST131 (**d**) with tip points coloured based on popPUNK cluster allocation. Lack of coloured point indicates that the isolate was assigned a novel cluster not present in the original global collection.

We next reconstructed a core-gene phylogeny using all 473 genomes from our collected isolates, 2776 assemblies from the global collection (selected to span the diversity of the collection) and 97 genomes from a previous study at QECH representing mostly invasive isolates from the same setting as our carriage collection. (Fig. 3). Malawian isolates were distributed throughout the tree, suggesting that Malawian ESBL-producing colonising strains represent a subsample of the global diversity of *E. coli *. Malawian isolates formed monophyletic clades within ST410, ST167 and ST131 (Fig. 3b, d), though in the case of ST131 (Fig. 3d) this included one isolate from the USA (year unknown).

**Fig. 4.**
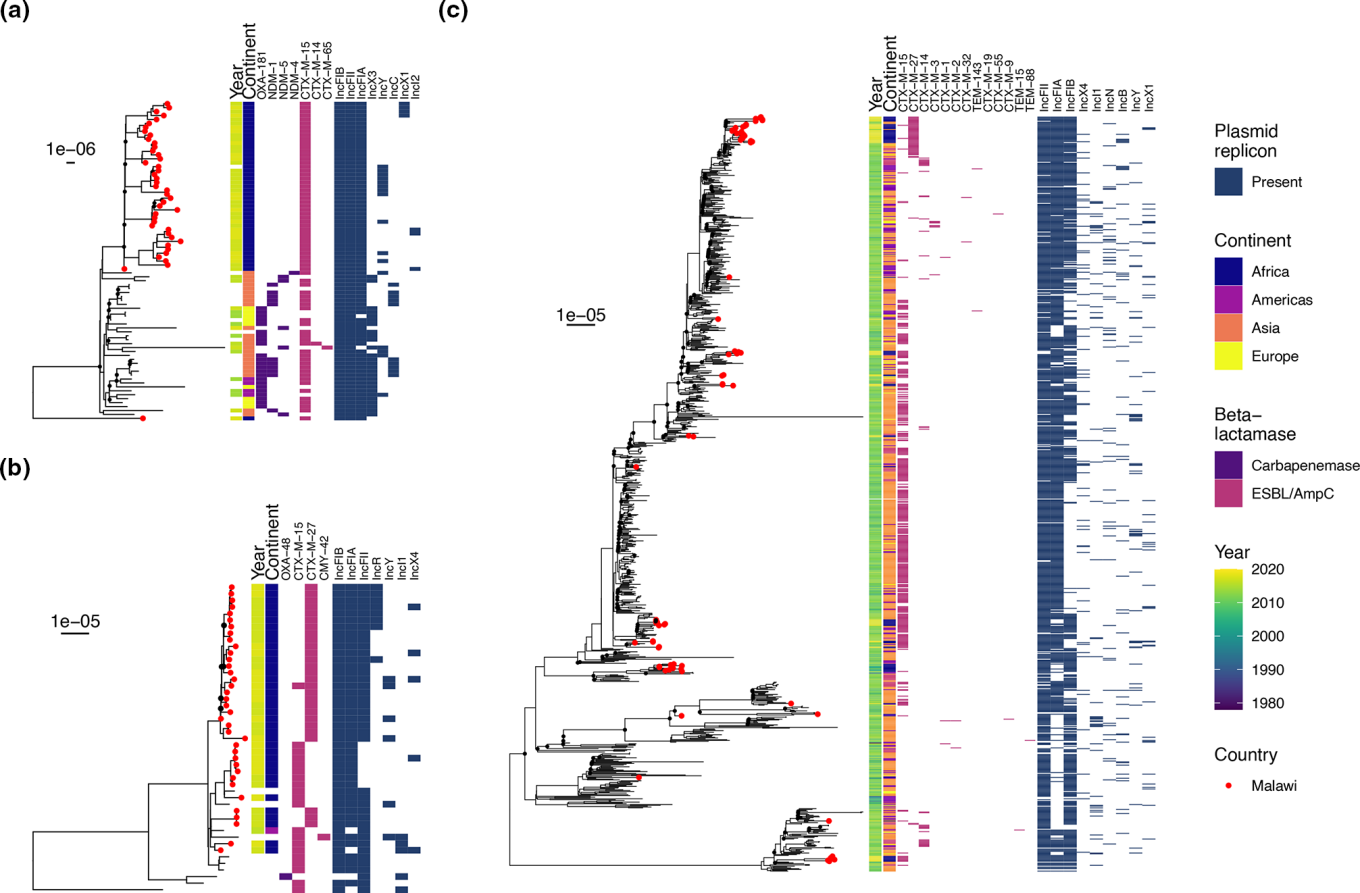
Subtrees of midpoint-rooted, maximum likelihood phylogenies of global *E. coli* ST410 (**a**) and ST167 (**b**) collections, and the global ST131 phylogeny (**c**) showing Malawian isolates (red tip points). Assemblies were constructed by mapping to ST-specific reference genomes. ESBL/CPE genes and plasmid replicons are shown. Bootstrap support of less than 95 % is shown by a black point at tree node. Full ST410 and ST167 global trees are shown in Figs S5 and S6, and subtree of monophyletic clade of exclusively isolates from Malawi in ST131 shown in Fig. S7.

To explore the genomic epidemiology of ST131, ST410 and ST167 with higher resolution, we reconstructed phylogenies by mapping each ST dataset to ST-specific reference genomes, incorporating 140 ST167, 371 ST410 and 919 ST131 genomes (Figs S5–S7 and Fig. 4), which confirmed our samples forming distinct monophyletic subclades in ST167 and ST410. The ST410 from our study (except for two isolates) are part of the globally distributed, often carbapenemase-associated B4/H24RxC lineage and formed a well-supported (>95 % ultrafast bootstraps) monophyletic clade within the B4/H24RxC lineage (Fig. 4a), with a median 35 (range 0–60) pairwise SNPs between the 43 Malawian isolates. All except one of our ST167 isolates comprised two lineages, one of which formed a well-supported (>95 % ultrafast bootstraps) monophyletic clade of only Malawian isolates (Fig. 4b) and a median 24 (range 0–66) pairwise SNPs between the 24 isolates; the other lineage included Malawian and global isolates. The Malawian ST131 isolates (*n*=64) were, in contrast, distributed throughout the ST131 phylogeny (Fig. 4c), though 14/64 isolates formed a monophyletic clade (Fig. S6) with median 75 (range 13–43) pairwise SNPs. In each of the three ST-specific collections, genomes from Africa were very poorly represented: two ST410 and no ST167 or ST131 were recorded as originating from Africa.

### Resistance and virulence determinants

**Fig. 5.**
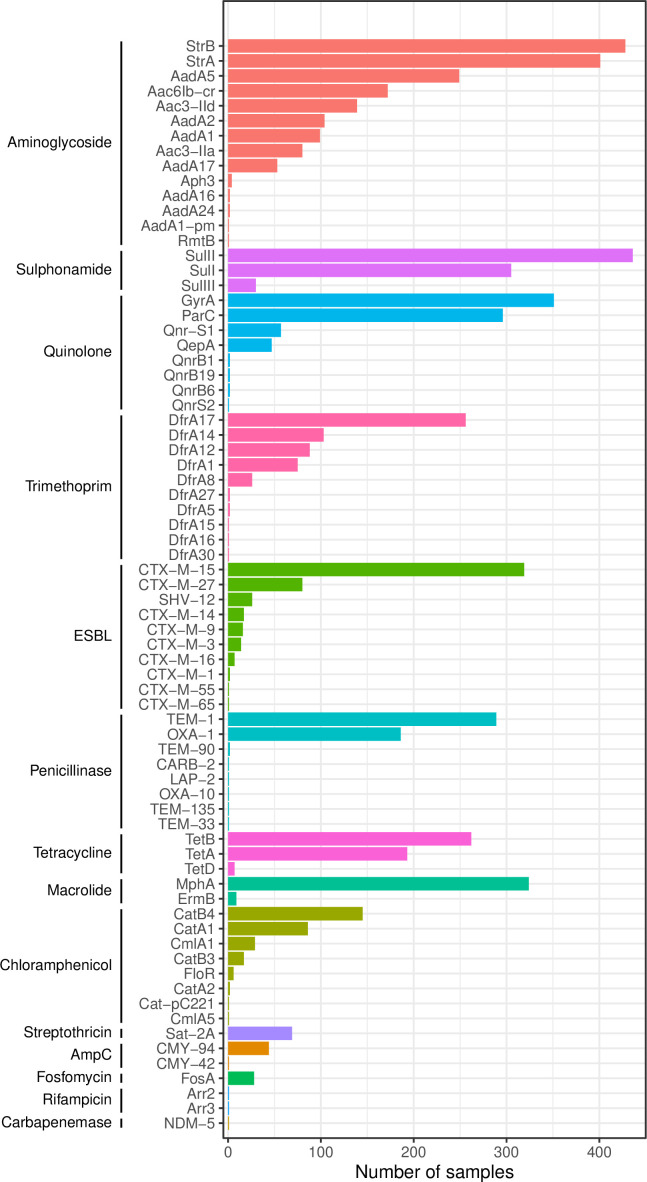
Distribution of identified antimicrobial resistance determinants

We identified a variety of AMR determinants in the isolates sequenced for this study (Fig. 5). One ST2083 isolate contained the carbapenemase encoding gene, *bla*_NDM-5_, which, in our short read *de novo* assembly, was co-located with an Inc-X3 containing contig, consistent with carriage on a plasmid, as we previously described [15]. Hybrid long read assembly revealed this isolate carried a plasmid almost identical to a previously described plasmid, pNDM5_020026 (Fig. S7; using the default BRIG cut off of 70 % similarity to display a sequence section as conserved).

This *bla*_NDM-5_-associated IncX3 plasmid is often associated with the ST410 B4/H24RxC lineage [9]. Accordingly we long read sequenced a representative of our ST410 isolates, which confirmed that it carried two of the four ST410 B4/H24RxC-associated plasmids, including the *bla*_CTX-M-15_ carrying pCTXM15_020026 plasmid, but lacks pNDM5_020026 (Fig. S8).

The remainder of the isolates without carbapenemases (*n*=472) carried at least one identified ESBL-encoding gene, consistent with the phenotype and most commonly *bla*_CTX-M-15_ which was present in 319/473 (67 %) of isolates. All other identified ESBL-encoding genes were members of the *bla*_CTX-M_ family except for *bla*_SHV-12_ which was identified in 26/473 isolates across six STs, but which was particularly common within ST656; all 17 ST656 isolates in the collection carried *bla*_SHV-12_ (Fig. S9).

Co-occurring determinants of resistance to aminoglycosides (99 % [472/473] of isolates), trimethoprim (97 % [459/473]), sulphonamides (99 % [468/473]) and quinolones (86 % [407/473]), were very common whilst genes conferring resistance to chloramphenicol were of lower frequency (52 % [248/473]). The most frequently identified quinolone resistance determinants were QRDR mutations (*gyrA* in 74 % [351/473] and *parC* in 63 % [296/473] isolates), but plasmid-mediated quinolone resistance determinants were also found (*qnrS* [12 %, 58/473], *qnrB* [1 % 6/473] and *qep* [10 %, 47/473]). The *gyrA* mutants were S83L (*n*=351), D87N (*n*=294) and *parC* mutants S80I (*n*=296) and S84G (*n*=6); co-occurrence of QRDR mutations and *qnr* genes was unusual (six isolates). Most (461/473 [97 %]) strains lacked the genes to classify them as any pathotype: 2/473 were identified as aEPEC/EPEC and 10/473 as EAEC (Fig. S3).

## Discussion

Understanding how the genomic epidemiology of *E. coli* in multiple LMICs differs from high income settings is essential to provide insight into local and global drivers of transmission. Our genomic analysis of the diversity of ESBL-producing *E. coli* in Blantyre, Malawi, enhances our understanding of the global ESBL *E. coli* genomic population structure by adding data from an under-sampled location. ESBL-producing *E. coli* colonising adults in Malawi represents the global diversity of the species with all major phylogroups, and 57 STs are represented.

Some global trends in AMR-associated *E. coli* ST are broadly reflected in Blantyre; for example ST131, the most frequently isolated invasive ST in many settings worldwide [48] (and frequently ESBL-producing), was the most commonly isolated ST, followed by the globally emergent AMR-associated ST410 and ST167 [8, 9]. By placing the Malawian isolates in a wider context we found diversity in the Malawian collection not captured in the curated species-wide Horesh collection [10]. Phylogroup distribution differs between our isolates and the Horesh isolates; phylogroup distribution has been shown to differ between continents, which may explain some of the diversity we describe in the isolates from Malawi [49]. There was a difference in relative prevalence of lineages as defined by popPUNK between Malawian and the global collection, with many Malawian isolates forming distinct clusters. Some of this may be due to a lack of diversity of ESBL-producing carriage isolates in the global collection; in the global collection most of these isolates were ST131. Both the core gene and map-to-reference phylogenies (using different context genomes) were consistent with local Malawian subclades in ST410 and ST167. This could be due to true locally circulating subclades or sampling biases in global *E. coli* collections either spatially (i.e. sampling high income settings) or particular lineages (e.g. sampling ST131 or other important pathogens) or resistance phenotypes (e.g. we have selected for ESBL producers). Only 246/10146 genomes in the global *E. coli* dataset used to build the curated collection in Horesh *et al*. were from the African continent [10] so it is possible (even likely) that apparent Malawian clades represent a lack of sampling elsewhere and are circulating regionally or more widely on the continent.

Scaling up of genomic surveillance in sSA with sampling of human, environmental and animal isolates can redress this imbalance, improve understanding of the global transmission of AMR *E. coli *, and should be an international priority for funders of future genomic epidemiology studies. Efforts to expand global collections (such as the species-wide collection used here) in an unbiased manner – with inclusion of ESBL-producing carriage isolates - as further genomes from low- and middle-income countries are sequenced will also be key to developing a comprehensive picture of local, national, and global scales of AMR transmission.

In Blantyre, as worldwide, *bla*_CTX-M_ is the dominant ESBL family, especially *bla*_CTX-M-15_. Amongst our isolates, which were selected based on phenotypic ESBL production, only one isolate carried a carbapenemase encoding gene: *bla*_NDM-5_. As observed commonly in collections of ESBL-producing bacteria dependent on mobile elements, aminoglycoside, trimethoprim, and sulphonamide resistance determinants were near-universal, and ciprofloxacin and chloramphenicol resistance determinants common. In terms of plasmids the co-occurrence of *bla*_CTX-M-15_ and IncF plasmids in our isolates reflects predominant global observations [50, 51].

This AMR-determinant distribution may be influenced by local antimicrobial pressures: carbapenem antimicrobials are at best sporadically available in QECH, but co-trimoxazole as CPT is widely used in this high HIV-prevalence setting as lifelong prophylaxis against infection in people living with HIV, as per World Health Organisation guidelines [17]. The ubiquity of genes conferring resistance to co-trimoxazole in this collection (i.e. *dfr, sul*) raises the possibility that use of CPT is creating selection pressure for other AMR-determinants in Blantyre. CPT has been shown to reduce mortality due to bacterial infection and malaria [17] in people living with HIV in high-burden settings. However, in an era of increasingly effective HIV treatment and increasing Gram-negative resistance it may be that a more nuanced approach to the deployment of CPT is needed.

ESBL-producing Gram-negative infections are an increasing clinical problem in Malawi, and there is a significant unmet need for access to carbapenem antimicrobials to treat them. Carbapenemase producing *Enterobacterales* including *E. coli* (*bla*_NDM-5_ in *E. coli* ST636) have recently been described in other regions of Malawi [52], and *bla*_NDM-5_ is increasingly being identified across sSA [53]. The presence of carbapenemases in this collection despite minimal local carbapenem use highlights the need for ongoing surveillance as these last-line antimicrobials are introduced. Globally, ST167 and ST410 include carbapenemase-associated lineages, but carbapenem-associated ST410 and 167 have not yet been observed in our setting. Amongst ST410, our isolates are however closely related to the globally distributed B4/H24RxC *bla*_NDM-5_/*bla*_OXA-181_ carbapenemase-associated lineage but lacked carbapenemase genes. Long read sequencing demonstrates however that the characteristic B4/H24RxC *bla*_NDM-5_-associated plasmid is almost identical to the plasmid observed in our collection in a different sequence type (ST2083), highlighting how important it is to introduce surveillance of emerging carbapenemase-expressing bacteria. Lack of carbapenemase encoding genes in our B4/H24RxC isolates could be due to lack of acquisition of harbouring plasmids, or due to repeated acquisition and subsequent loss events given the current absence of carbapenem selection pressure. If the latter, it would suggest the fitness trade-off to harbour the plasmid might select against maintenance in the absence of the selection pressure within this lineage for now. Availability of carbapenems is predicted to change.

Chloramphenicol has historically been a first-line treatment for severe febrile illness in Malawi [54] but increasing numbers of resistant isolates causing infections has curtailed its use in favour of ceftriaxone [55]. Chloramphenicol resistance determinants were however absent in 48 % of our samples; indicating that chloramphenicol could still have a role to play as a reserve agent in the treatment of ESBL-* E. coli *, but this approach would require quality assured diagnostic microbiology services to support it.

There are limitations to our study. Some participants provided multiple samples, so it is possible that this introduced bias into the collection. Our study was predominantly based at a single urban hospital, over a period of around only 2 years so although it contributes valuable genomes from an under sampled region and population, the extent to which these genomes are representative of other areas on Malawi is unclear. Furthermore, our samples were cultured on ESBL-selective media, meaning we provide a snapshot of ESBL-positive *E. coli* genomic diversity in Malawi, rather than of *E. coli* infecting humans as a whole, which contrasts with the Horesh study, which was a combination of collections that had different selection criteria, leading to its own biases. We classified Horesh samples as being ‘invasive’ or ‘stool’ but there was a high proportion of missing data and some of these ‘stool’ isolates may have been enterotoxigenic or enteropathogenic *E. coli* (ETEC or EPEC) – though the Horesh *et al.* publication estimates that these pathotypes were very unusual (<5 %), which justifies our approach [10].

In conclusion, we find that the diversity of ESBL *E. coli* from Blantyre includes globally distributed high-risk clones and broadly reflects the global population structure. The occurrence of distinct monophyletic subclades when assessed in the context of a large recent data collection highlights the need for further targeted sequencing of isolates from Malawi and sSA to understand local, regional, and global *E. coli* circulation. Carbapenemase-associated lineages reported elsewhere in the world are present in Malawi, but currently still lack carbapenem resistance determinants. However, the presence of carbapenemase-encoding plasmids in our collection highlights that it is likely only a matter of time before either these lineages are selected for and evolve into high-risk clones or carbapenemase genes on mobile genetic elements are transmitted to a high-risk clone. There is a critical need for both robust stewardship strategies and for ongoing genomic surveillance with rapid reporting, as these agents are introduced.

## Supplementary Data

Supplementary material 1
