# Supplementary material for: Genomic analysis of extended-spectrum beta-lactamase (ESBL) producing Escherichia coli colonising adults in Blantyre, Malawi reveals previously undescribed diversity
Source: Microb Genom. 2023 Jun 14;9(6):mgen001035. doi: 10.1099/mgen.0.001035 (PMC10327512; doi:10.1099/mgen.0.001035)
Supplement: Supplementary material 1 [file mgen-9-1035-s001.pdf]

Genomic analysis of extended-spectrum beta-lactamase (ESBL) producing *Escherichia coli* colonising adults in Blantyre, Malawi reveals previously undescribed diversity

Supplementary material

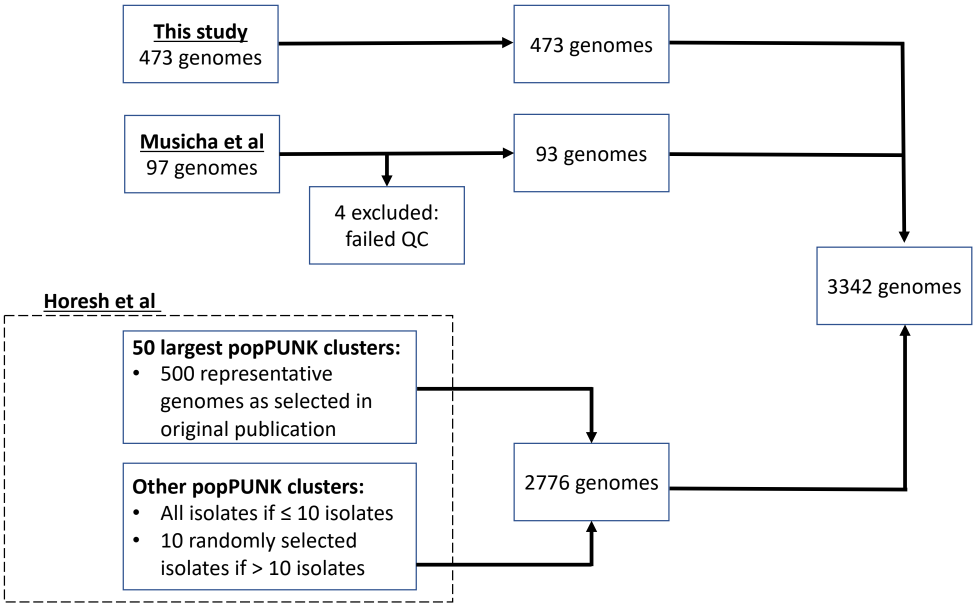

Supplementary Figure 1: Flowchart of included contextualising isolates.

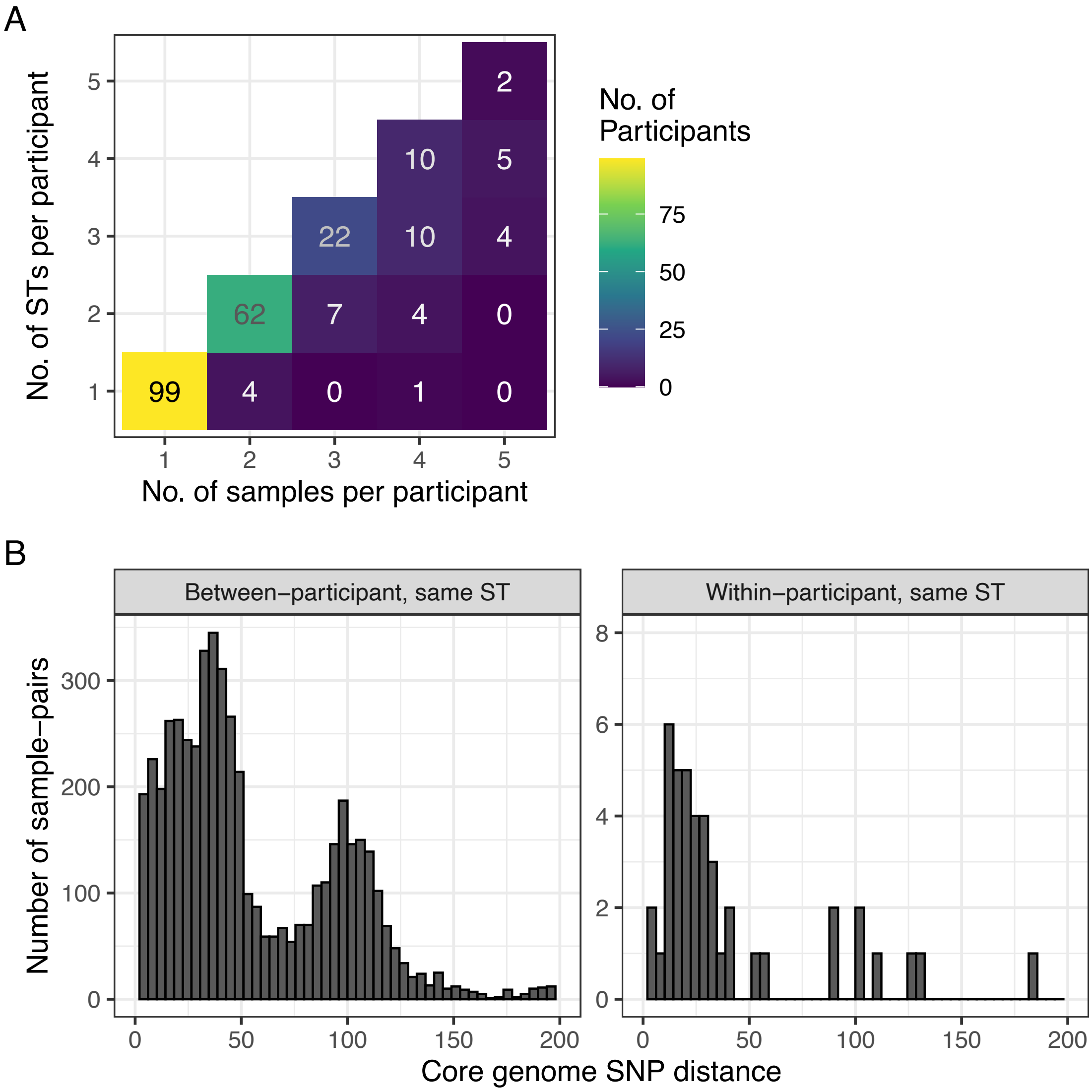

Supplementary figure 2: Describing within-participant diversity. A: Heatmap showing number of participants with a given number of samples and number of STs per participant. Most (195/230, 85%) participants do not contribute samples with a duplicated ST. B: Histogram of distribution of pairwise core genome SNP distance, considering only sample pairs of the same ST, and stratified by whether sample pair is between- or within-participant. Distributions are similar, justifying keeping all samples in the analysis. Histogram has bin size 4 SNPs and x-axis is restricted to ≤ 200 SNPs to show closely related isolates.

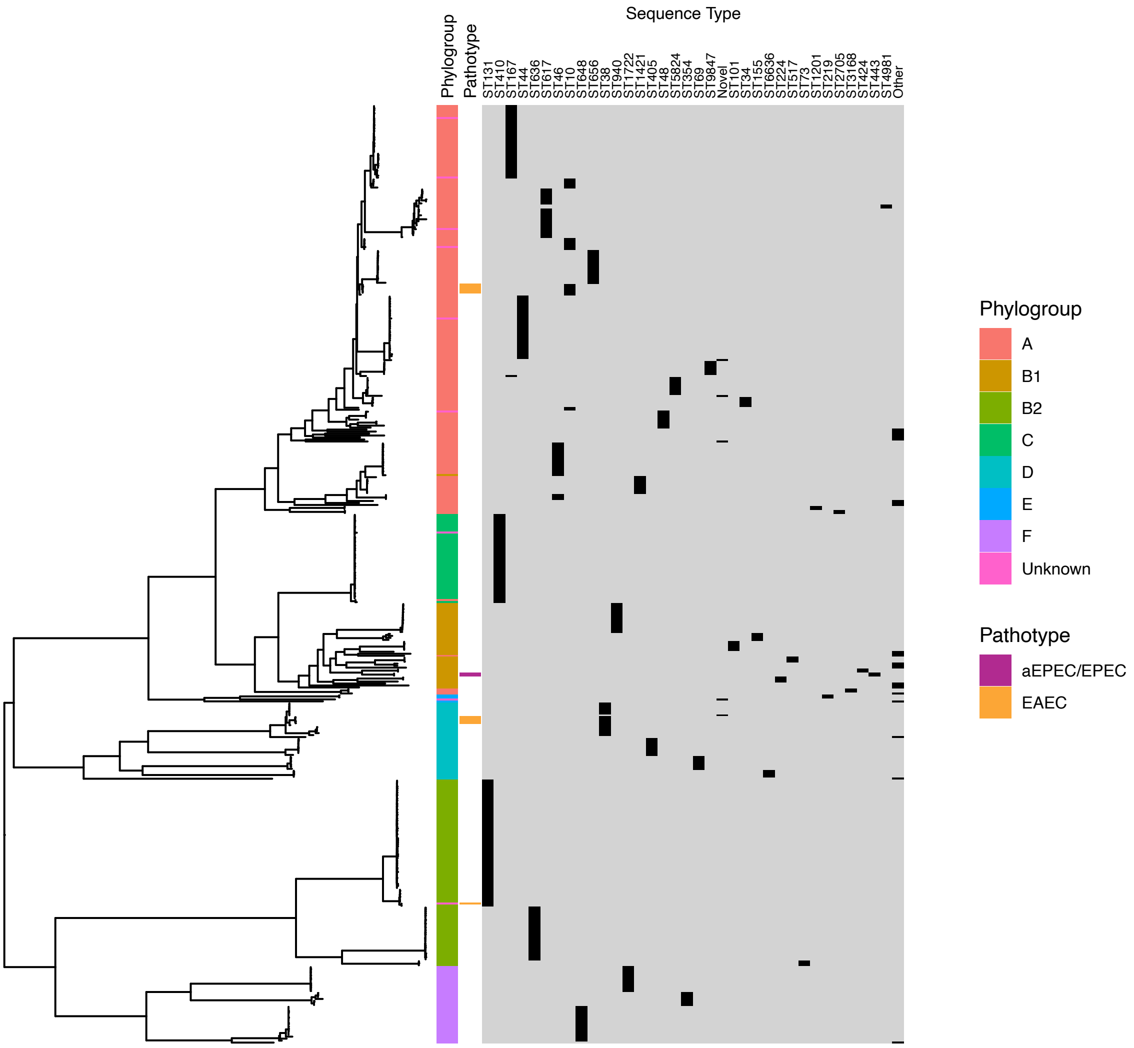

Supplementary Figure 3: Phylogeny of E. coli from this study. Maximum-likelihood midpoint-rooted phylogeny inferred using the general time-reversible nucleotide substitution model with FreeRate site heterogeneity with five parameters, as selected by IQTREE ModelFinder module. Phylogroups, STs and pathotypes shown.

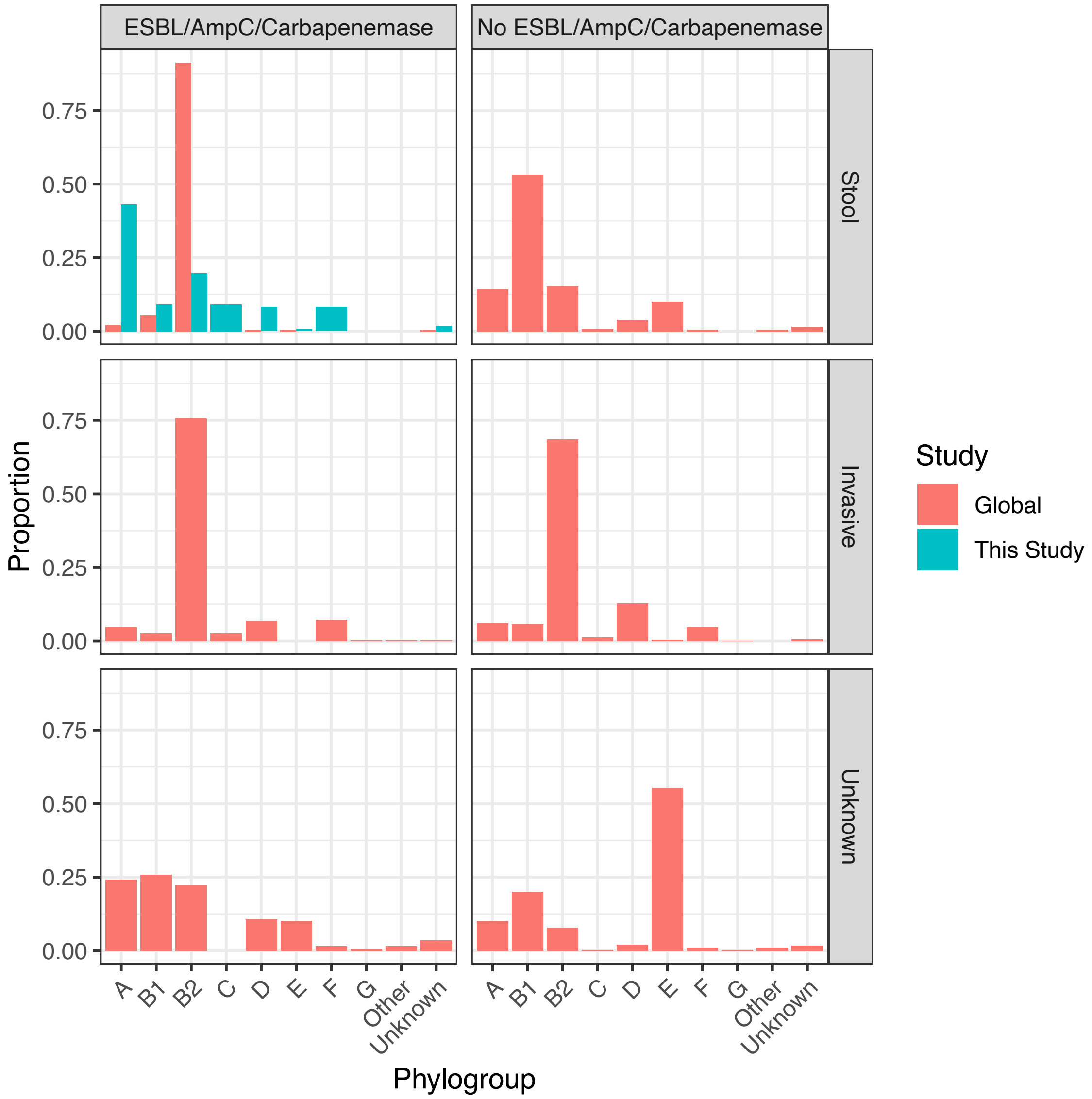

Supplementary Figure 4: Comparison of phylogroup distribution between this study and the global collection of *E. coli* isolates, stratified by source (from stool, invasive sample, or unknown) and presence of gene encoding for ESBL, AmpC or carbapenemase. Proportions shown are for the stratified groups so (for example) the proportion of phylogroup A isolates in the top left panel is the proportion of ESBL/AmpC/carbapenemase-encoding isolates from stool that fall into that phylogroup.

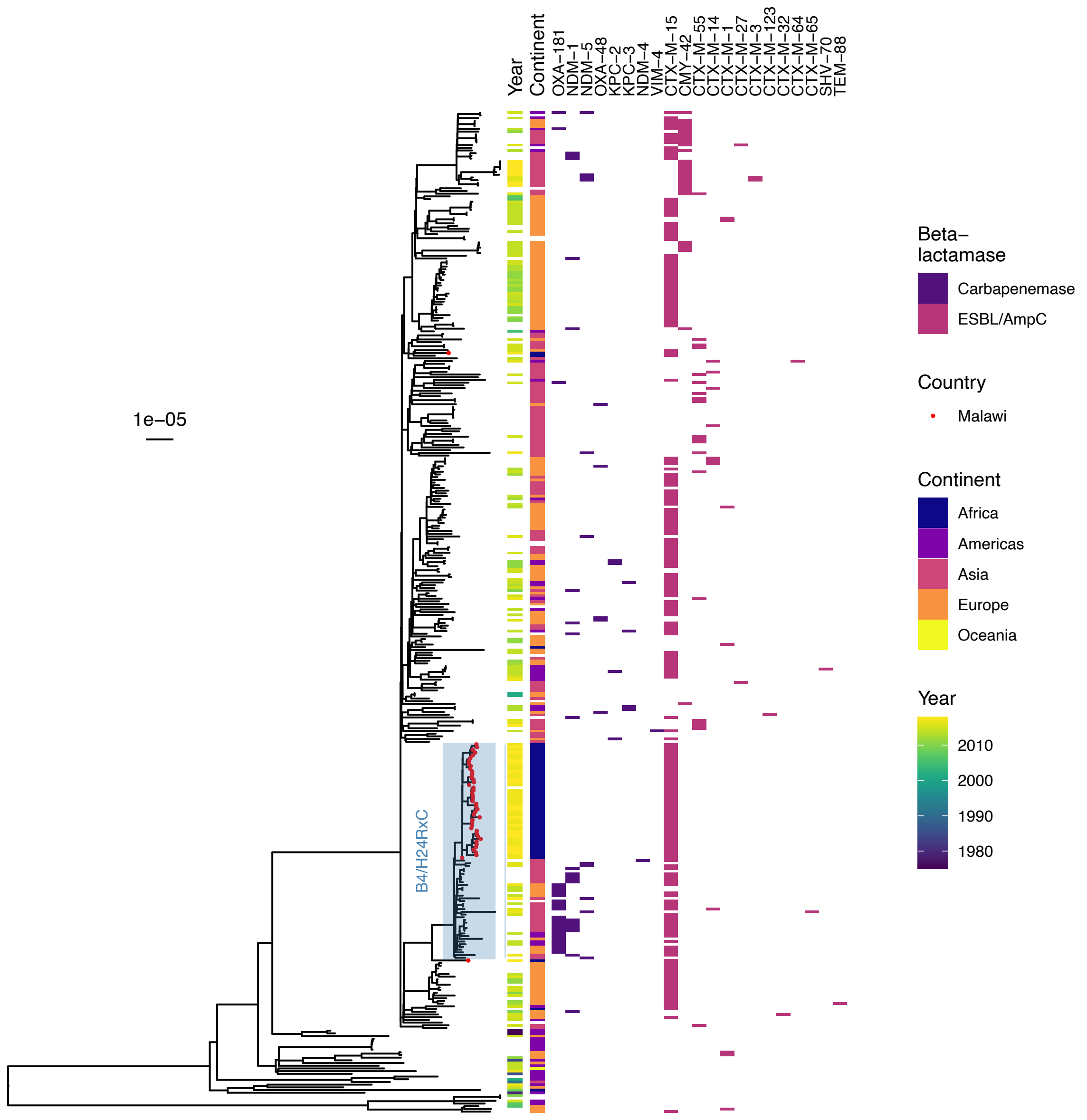

Supplementary Figure 5: Midpoint-rooted maximum likelihood phylogeny of global E. coli ST410, with assemblies obtained by mapping to reference. ESBL/CPE genes and plasmid replicons are shown. Blue shaded area shows the carbapenemase-associated B4/H24RxC lineage – this area is expanded in Figure 4.

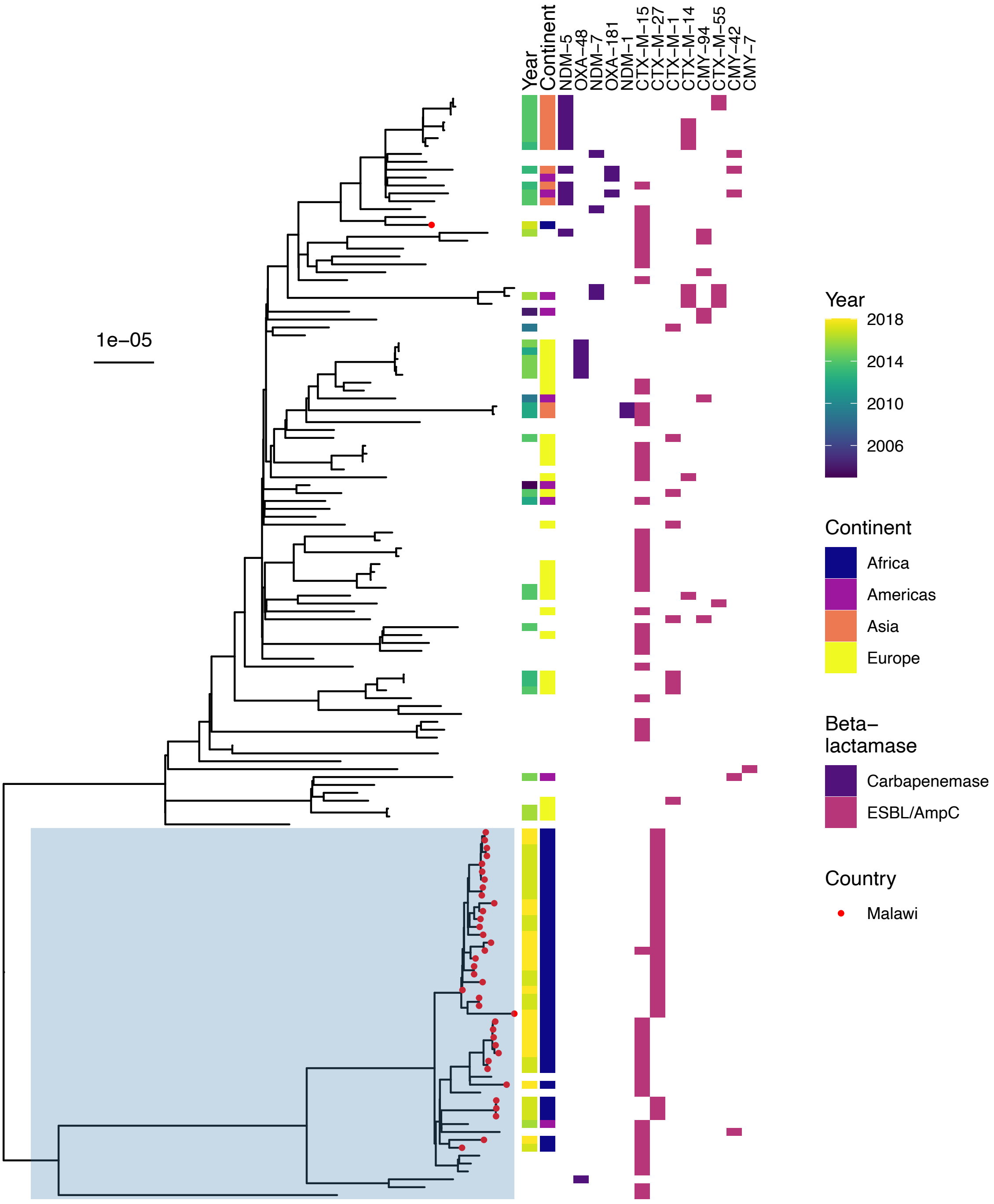

Supplementary Figure 6: Midpoint-rooted maximum likelihood phylogeny of global *E. coli* ST167, with assemblies obtained by mapping to reference. ESBL/CPE genes and plasmid replicons are shown. Blue shaded area shows the area that is expanded in the subtrees in Figure 4.

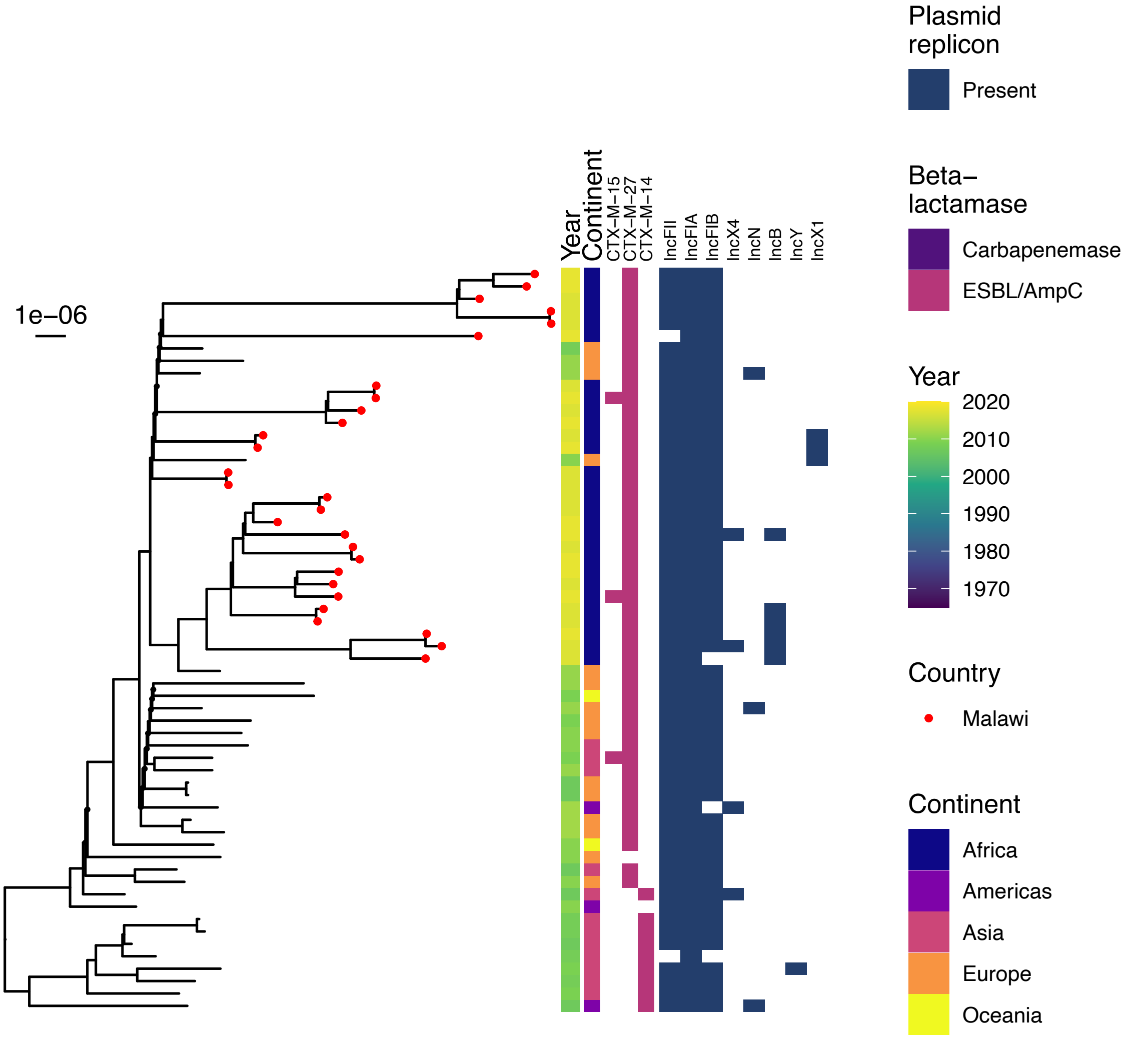

Supplementary Figure 7: Subtree of midpoint-rooted maximum likelihood phylogeny of global ST131 *E. coli*, showing area in which isolates from our study form a monophyletic clade.

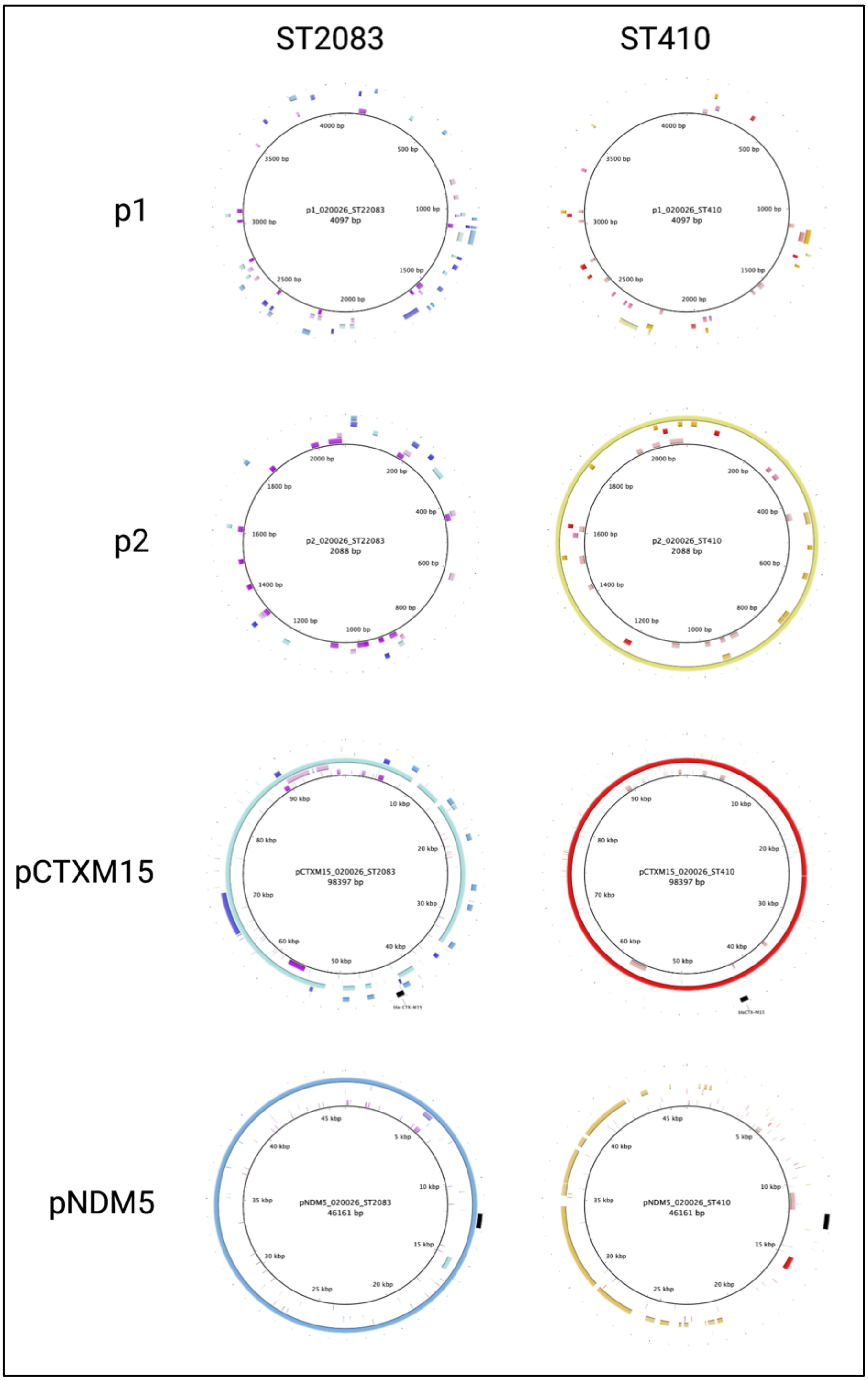

Supplementary Figure 8: Blast Ring Image Generator (BRIG) comparison of the plasmids present in the ST410 B4/H24RxC MDR isolate 020026 from Feng at al[8] with the ST2083 (left panels) and ST410 (right panels) assemblies from our study. Inner to outer ring show the different plasmids from the respective isolate (ST2083 or ST410). Whilst ST410 seems to share the identical plasmid p2 (right panel second row) and the blaCTX-M-15 encoding plasmid (right panel third row), the bla-NDM-5 encoding plasmid is only partially conserved; of particular importance, the bla-NDM-5

gene (indicated as black box outside all rings) is not present, as predicted by ariba. On the contrary, the ST22083 isolate has only partial similarity with the bla-CTX-M-15 encoding plasmid (third row, left column) but the completely conserved bla-NDM-5 encoding plasmid, including the NDM-5 gene (left panel, row four).

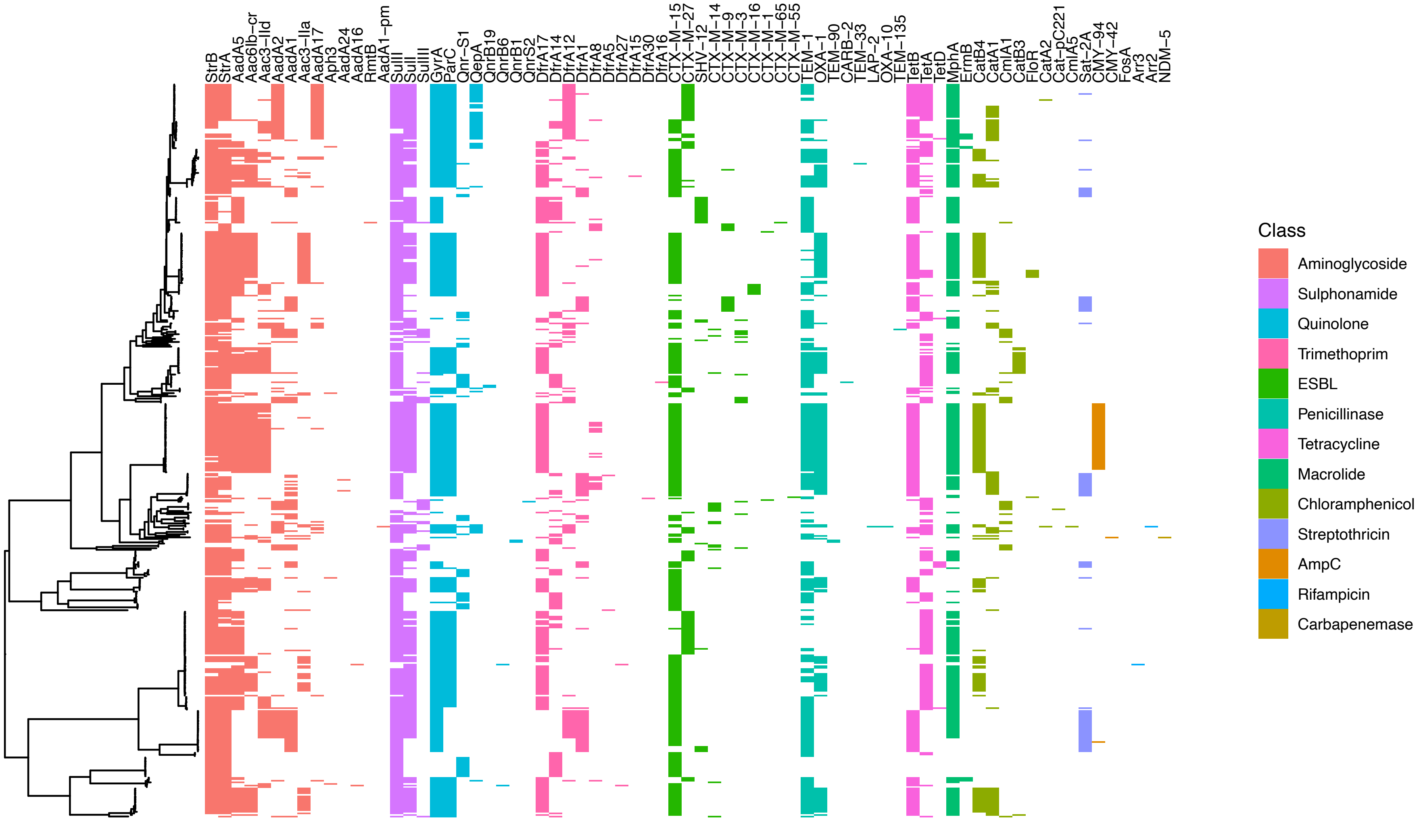

Supplementary Figure 9: Antimicrobial resistance determinants mapped back to core gene tree

Supplementary Table 1: *E. coli* pathotype definitions

**Definition**	**Pathotype**
Presence of any Shiga toxin gene	STEC (Shiga toxin producing *E. coli*)
Presence of *eae*	aEPEC/EPEC ([atypical] Enteropathogenic *E. coli*)
Presence of Shiga toxin plus *eae*	EHEC (Enterohaemorrhagic *E.coli*)
Presence of *aatA or aggR or aaiC*	EAEC (Enteroaggregative *E. coli*)
Presence of *est* or *elt*	ETEC (Enterotoxigenic *E. coli*)
Presence of pINV plasmid	EIEC (Entero invasive *E. coli*)
